# Real-world adverse event profile and signal characteristics of bevacizumab in glioma: a FAERS-based disproportionality analysis

**DOI:** 10.3389/fphar.2025.1705451

**Published:** 2026-01-13

**Authors:** Mingyue Gao, Qiao Chen, Hengheng Zhang, Yi Tian, Zhiguang Fu, Maohui Yan, Chen Liu

**Affiliations:** Department of Radiotherapy, Air Force Medical Center, The Fourth Military Medical University, PLA, Beijing, China

**Keywords:** adverse events, bevacizumab, disproportionality analysis, FAERS, glioma, pharmacovigilance, population heterogeneity

## Abstract

**Background:**

Bevacizumab is a critical anti-angiogenic therapy for glioma, but its real-world safety profile requires comprehensive characterization beyond clinical trials to effectively manage treatment risks.

**Methods:**

This pharmacovigilance study analyzed adverse event reports from the FDA Adverse Event Reporting System (FAERS) for glioma patients receiving bevacizumab. A disproportionate analysis using multiple analytical methods was conducted to identify significant safety signals. Subgroup analyses stratified by gender and age were performed to explore population heterogeneity.

**Results:**

Bevacizumab-related AEs involved multiple system organ classes, with particularly prominent disproportionality signals for vascular disorders, especially hypertension, proteinuria and thromboembolic events such as pulmonary embolism. Most AEs occurred within the early treatment period, but late-onset events, including tumor progression, still accounted for a notable proportion. Subgroup analysis indicated that male patients were at higher risk of overall bleeding and thrombotic events, whereas female patients more frequently reported cognitive impairment and showed stronger signals for severe bleeding subtypes such as intracranial hemorrhage, suggesting sex-specific heterogeneity across bleeding phenotypes. Middle-aged patients bore the greatest burden of reported AEs.

**Conclusion:**

This study delineates the adverse event spectrum of bevacizumab in patients with glioma, confirming prominent vascular and renal toxicities and revealing sex- and age-related differences in safety profiles. The findings highlight the need for heightened surveillance of vascular and renal events following bevacizumab exposure and provide hypothesis-generating evidence to inform future prospective studies.

## Highlights

- This study characterizes 20-year trends and distribution patterns of bevacizumab-related AEs in glioma.
- Fluctuations in AE reports coincide with FDA indication and regulatory changes.
- The reporting proportion in North America is markedly higher than in other regions.
- Patients aged 45–64 years constitute a high-risk group for AEs.
- These findings provide real-world evidence for risk identification and pharmacovigilance of bevacizumab.

## Introduction

Glioma is the most prevalent primary malignant brain tumor of the central nervous system, believed to originate from neural stem or progenitor cells with oncogenic alterations, accounting for approximately 81% of all primary malignant brain tumors (Weller et al., 2024; Xu et al., 2020). In adults, the annual incidence of glioma is 5.6 per 100,000, making it one of the most common central nervous system malignancies. The prognosis for malignant glioma is extremely poor, with a 5-year survival rate typically below 5% (Zhong et al., 2023). This dire prognosis imposes significant psychological and economic burdens on patients and their families and exerts substantial public health and financial pressures on healthcare systems (Zhong et al., 2023). Current primary treatment strategies for glioma include surgical resection, radiotherapy, and chemotherapy, which have shown limited efficacy in slowing disease progression (Xu et al., 2020; Yang et al., 2022). Recently, advancements in molecular biology and tumor immunology have led to targeted therapies and immunotherapies entering clinical trial phases, showing promise in some patients. However, long-term clinical practice indicates that while targeted therapies offer certain benefits, associated adverse events (AEs) limit their use and may even lead to treatment discontinuation in some patients (Lombardi et al., 2019). Thus, achieving a balance between efficacy and toxicity remains a critical clinical challenge in the treatment of glioma.

Bevacizumab (BEV), a humanized monoclonal antibody, inhibits tumor angiogenesis by specifically binding to vascular endothelial growth factor (VEGF) and blocking its signaling pathway (Casadei-Gardini et al., 2023; Zheng et al., 2023; Rimini et al., 2022). In 2009, the U.S. Food and Drug Administration (FDA) granted accelerated approval for its use in recurrent glioblastoma (GBM), marking a milestone in the application of anti-angiogenic therapy within neuro-oncology (Cohen et al., 2009). Clinically, BEV reduces vascular permeability and alleviates cerebral edema, thereby mitigating neurological symptoms and extending progression-free survival (PFS) in selected patients (Detti et al., 2021; Tsien et al., 2023). However, its benefit in prolonging overall survival (OS) has not been consistently demonstrated, and its therapeutic role remains a matter of debate. More critically, prolonged administration introduces new challenges. Studies suggest that BEV may induce a hypoxic microenvironment, driving tumor cells to adopt VEGF-independent pathways that enhance invasiveness and foster resistance (Shimizu et al., 2019; Chandra et al., 2020). In addition, treatment is frequently complicated by adverse effects, including hypertension, proteinuria, thromboembolic events, and hemorrhage (Wichelmann et al., 2021; Gu et al., 2022; Yang et al., 2025). These toxicities not only restrict its broader clinical application but also adversely affect patients’ quality of life and adherence to therapy.

In recent years, research on AEs associated with BEV has increased; however, a systematic description of its safety profile in the glioma population remains lacking. While previous randomized controlled trials (RCTs) have provided preliminary evidence regarding the efficacy and partial safety of BEV, their findings often do not fully represent the risk profile of real-world patients due to strict inclusion criteria and limited follow-up periods (Sferruzza et al., 2024; Camerlingo, 2021). RCTs typically exclude patients with complex comorbidities or poor prognosis, potentially underestimating the incidence of AEs in the general population. Moreover, existing pharmacovigilance studies on BEV’s safety are mostly based on a broad cancer population and lack a specific focus on glioma patients (Wang et al., 2024). This results in significant heterogeneity among different studies, making it challenging to directly compare the incidence, types, and risk factors of AEs. Particularly concerning demographic differences, current research has yet to clearly elucidate how factors such as gender and age influence BEV-related AEs (Wang et al., 2024; Fukuda et al., 2023). Furthermore, some late-onset events may not be adequately captured, limiting the completeness and representativeness of existing studies. Therefore, systematically and comprehensively exploring the spectrum of AEs associated with BEV in glioma patients and identifying high-risk populations remains an urgent scientific issue.

The monitoring of AEs is a critical component in ensuring the safety of clinical drug use. Once new drugs are widely applied in clinical settings, their real-world safety profile often diverges from that observed in clinical trials. This is particularly true for antineoplastic agents, which, due to their unique mechanisms of action, prolonged treatment durations, and the complex baseline conditions of patients, are more prone to inducing a variety of AEs. Existing literature has highlighted that the types of AEs associated with BEV are extensive, affecting multiple systems, including the vascular, renal, gastrointestinal, and nervous systems (Wichelmann et al., 2021; Gu et al., 2022). Some of these AEs can manifest early on, such as hypertension and proteinuria, while others may emerge during long-term treatment, including thrombosis, hemorrhage, and even tumor progression (Kim et al., 2024; Fukuda et al., 2023). For patients with glioma, whose disease course is inherently complex, distinguishing between the natural progression of the disease and drug-related AEs presents a significant research challenge. Additionally, the variation in risk across different populations has not been systematically summarized, leaving clinicians without clear criteria for risk stratification in practice. Therefore, a comprehensive understanding of the spectrum of AEs associated with BEV in glioma populations is crucial for optimizing risk assessment and clinical management.

Previous work has also evaluated the safety of bevacizumab in glioblastoma using the FAERS database. Qu et al. (2025) analyzed bevacizumab-related adverse event reports from 2004 to 2023 and compared them with those of temozolomide and lomustine, identifying prominent signals for vascular and renal toxicities, with relatively milder hematologic toxicity. Building on this, the present study expands the target population from glioblastoma to the full spectrum of gliomas, extends the observation period to the fourth quarter of 2024, and places particular emphasis on population heterogeneity (sex, age, and reporter type) as well as the temporal distribution of adverse events, thereby providing a more comprehensive characterization of the safety profile of bevacizumab. The primary objective is to delineate the adverse event landscape of bevacizumab in patients with glioma, with a focus on signal strength and intergroup differences. By synthesizing real-world adverse event reports, this study aims to identify both common and serious toxicities and to explore how adverse event patterns vary across patient subgroups. Scientifically, these findings offer empirical evidence to support future pharmacovigilance and mechanistic research; clinically, they are expected to inform risk stratification and individualized management, assist clinicians in balancing efficacy and safety, improve treatment adherence and overall outcomes, and ultimately provide a data-driven basis for optimizing the rational use of bevacizumab and for the development of future personalized treatment strategies and clinical guidelines in glioma.

## Materials and methods

### Data sources

This study utilizes the U.S. Food and Drug Administration Adverse Event Reporting System (FAERS) database (https://fis.fda.gov/extensions/FPD-QDE-FAERS/FPD-QDE-FAERS.html), with data downloaded on 15 January 2025, covering all publicly available data from the first quarter of 2004 to the fourth quarter of 2024. The FAERS data comprises seven tables: demographics and administrative information (DEMO), adverse reactions (REAC), patient outcomes (OUTC), drug information (DRUG), therapy dates and timing (THER), report sources (RPSR), and indications information (INDI).

Following FDA-recommended methods and previous studies (Shu et al., 2022; Wang et al., 2023; Cui et al., 2023), data were stratified based on PRIMARYID, CASEID, and FDA_DT fields: retaining only the most recent record by FDA_DT; if CASEID and FDA_DT are identical, the record with the highest PRIMARYID is retained. All deduplication procedures were executed in an R 4.4.1 environment, with an example of deduplication rules presented in Table 1.

**TABLE 1:** Example of duplicate report removal rules in FAERS database.

CASEID	FDA_DT	PRIMARYID	DELETE OR SAVE
5657190	20061207	43682654	DELETE
5657190	20061225	43693684	SAVE
5660307	20080302	53651284	DELETE
5660307	20080302	54635428	SAVE

Data source FAERS, database, 2004Q1–2024Q4.

AEs were standardized using MedDRA version 27.1 (released in September 2024), which encompasses system organ class (SOC), high-level group terms (HLGT), high-level terms (HLT), preferred terms (PT), and low-level terms (LLT).

### Data processing and selection

**FIGURE 1:**
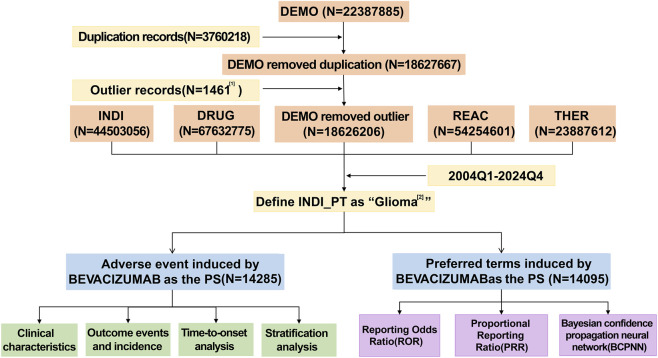
Technical Roadmap. Note: (1) Individuals with ages less than 0 years and greater than 120 years, as well as those with weights exceeding 400 kg, are classified as outliers and are excluded from the analysis. (2) A total of 28 MedDRA PT are included, as detailed in Supplementary Table S1. DEMO refers to Demographics and Administrative Information; INDI denotes Indications Information; DRUG pertains to Drug Information; REAC is Reaction Information; THER represents Therapy Dates and Timing.

AE reports from the first quarter of 2004 through the fourth quarter of 2024 were retrieved from the FAERS database, yielding a total of 18,626,206 records. According to the FDA Drug Portal, BEV was approved in February 2004; therefore, the analysis was restricted to reports submitted between 2004Q1 and 2024Q4. Drug names were standardized using the WHO Drug Dictionary (September 2024 edition). After filtering for cases in which BEV was identified as the primary suspect (PS) drug and the indication was glioma, 14,285 reports were included (14,095 PS records). Glioma-related indications were defined according to MedDRA PTs, encompassing 28 diagnostic categories (Supplementary Table S1). The workflow for data extraction and analysis is presented in Figure 1.

Patients meeting the inclusion criteria and exposed to BEV were further analyzed across several variables: gender, age, body weight, reporting year, SOC, reporter occupation, continent of origin, clinical outcome, and time to onset of AE. Time to onset was calculated as the interval between the event onset date (EVENT_DT in the DEMO dataset) and the treatment start date (START_DT in the THER dataset). Missing values were coded as “not specified [NS].” Age was categorized into four groups: <18, 18–44, 45–64, and ≥65 years; weight was stratified as < 80 kg and >80 kgg, in line with the *Exposure Dose Guidance for Body Weight* that uses 80 kg as the default adult body weight and with prior FAERS-based pharmacovigilance studies (Meng et al., 2025; Hu et al., 2024a). This weight stratification was used solely for descriptive statistics and subgroup comparisons, and not for inferential analyses. Reporter occupation was classified as (1) consumer (CN)-related reporters (CN, LW: lawyer, SALES: salesperson) and (2) healthcare professionals (RN: registered nurse, PH: pharmacist, MD: physician, OT: other health professional). Outcomes were grouped as death (DE), disability (DS), hospitalization (HO), life-threatening events (LT), other serious, required intervention (RI), and congenital anomaly (CA).

### Descriptive statistics and disproportionality analysis

Initially, descriptive statistical methods (frequencies, percentages, interquartile range [IQR]) were used to summarize the demographic characteristics, RPSR, and outcomes of AE reports associated with BEV. Missing values were consistently coded as ‘unknown’ and excluded from percentage calculations. In subgroup analyses by gender, age, reporter occupation, etc., items with fewer than 30 reports (n < 30) were excluded to control for statistical instability. Subsequently, a disproportionality analysis was conducted using a 2 × 2 contingency table (a: drug-AE, b: drug-non-AE, c: non-drug-AE, d: non-drug-non-AE) to calculate association strength indicators and evaluate the strength of association between BEV and specific AEs.

### Three common signal detection methods were utilized

1. Reporting odds ratio (ROR): ROR = (a/c)/(b/d) = (a × d)/(b × c). ROR is suitable for binary outcome analysis in large sample data. A signal is identified when ROR >1 and the lower limit of the 95% confidence interval (CI) > 1 (WHO-UMC standard) (Li et al., 2008). This method is sensitive to rare events but may be affected by data sparsity.
2. Proportional reporting ratio (PRR): PRR = [a/(a+b)]/[c/(c + d)]. PRR assesses statistical significance through the ratio of drug-AE report proportion to non-drug-AE report proportion combined with a χ^2^ test (degrees of freedom = 1). A positive signal is identified when PRR >2 and χ^2^ > 4 (Evans standard) (Evans et al., 2001). This method is more stable with small-sample data but less sensitive to low-frequency events.
3. Bayesian confidence propagation neural network (BCPNN): It calculates the Information Component (IC) and its lower limit (IC_025_ [Information Component 025]), identifying a signal when IC_025_ > 0 (WHO-UMC method) (Bate et al., 2002). BCPNN quantifies signal strength through a Bayesian framework by comparing prior and posterior distributions, making it particularly suitable for handling sparse data and complex association networks despite high computational complexity. For signal assessment, in addition to classical pharmacologic adverse events (e.g., hypertension, proteinuria, bleeding, thrombosis), selected MedDRA terms reflecting overall safety outcomes or use patterns (e.g., “death”, “disease progression”, “off-label use”, “product use issue”) were retained. Although not pharmacologic reactions in the strict sense, these terms are treated in pharmacovigilance frameworks as potential safety-risk signals and were therefore included in descriptive analyses, whereas primary signal screening focused on pharmacologic events.

The choice among these methods is based on their complementarity: ROR is widely recognized as a classical method, PRR enhances robustness through proportional comparison, and BCPNN compensates for the limitations of the former two methods concerning data sparsity via probabilistic modeling. If any method reaches the threshold, it is considered a potential drug-related REAC signal. This study uses ROR as the primary analysis indicator, with PRR and BCPNN serving as validation indicators. To control for multiple comparison errors, the Benjamini–Hochberg method was applied for false discovery rate correction with a significance threshold set at *p* < 0.05 (two-tailed test). Additionally, sensitivity analysis was performed by excluding cases where ‘the indication was atypical glioma’ to ensure that specific indication populations do not influence signals.

**FIGURE 2:**
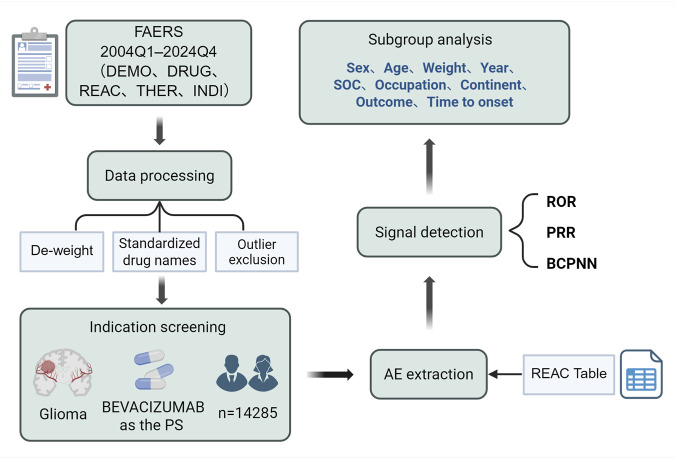
Overall analysis workflow.

All analyses were conducted in R version 4.4.1 software environment using packages such as PhViD (v2.1.0), epiR (v2.0), and dplyr (v1.1.4). Refer to Table 2 for the structure of contingency tables and Table 3 for detailed information on disproportionality analysis methods and thresholds; Figure 2 illustrates the overall analytical workflow.

**TABLE 2:** Fourfold table for disproportionality analysis.

Item	Target adverse events	All other adverse events	Total
Target drugs	a	b	a+b
All other drugs	c	d	c + d
Total	a+c	b + d	a+b + c + d

Data source FAERS, database, 2004Q1–2024Q4.

**TABLE 3:** Principles of disproportionality analysis and criteria for signal detection.

Method	Calculation formula	Inclusion standard of positive signal
ROR	ROR = (a/c)/(b/d) = ad/bc SE (lnROR) = √(1/a +1/b + 1/c + 1/d) 95%CI = lnROR ±1.96 × SE (lnROR)	Lower limit of 95%CI > 1
PRR	PRR = [a/(a+b)]/[c/(c + d)] SE (lnPRR) = √(1/a - 1/(a+b) + 1/c - 1/(c + d)) 95%CI = lnPRR ±1.96 × SE (lnPRR) χ^2^ = (ad - bc)^2^ (a+b + c + d)/((a+b) (c + d) (a+c) (b + d))	PRR ≥2 and χ^2^ ≥ 4
BCPNN	IC = log2 [(a/(a+b))/((a+c)/(a+b + c + d))] IC025 = IC - 1.96 × √[1/(a+b) + 1/(c + d)]	IC025 > 0

a = number of reports for target drug–target adverse event; b = number of reports for target drug–all other adverse events; c = number of reports for all other drugs–target adverse event; d = number of reports for all other drugs–all other adverse events. ROR, reporting odds ratio; PRR, proportional reporting ratio; BCPNN, bayesian confidence propagation neural network; IC, information component; IC025 = lower limit of the 95% confidence interval of IC., Data source; FAERS, database, 2004Q1–2024Q4.

## Results

### Baseline characteristics of AE reports

**FIGURE 3:**
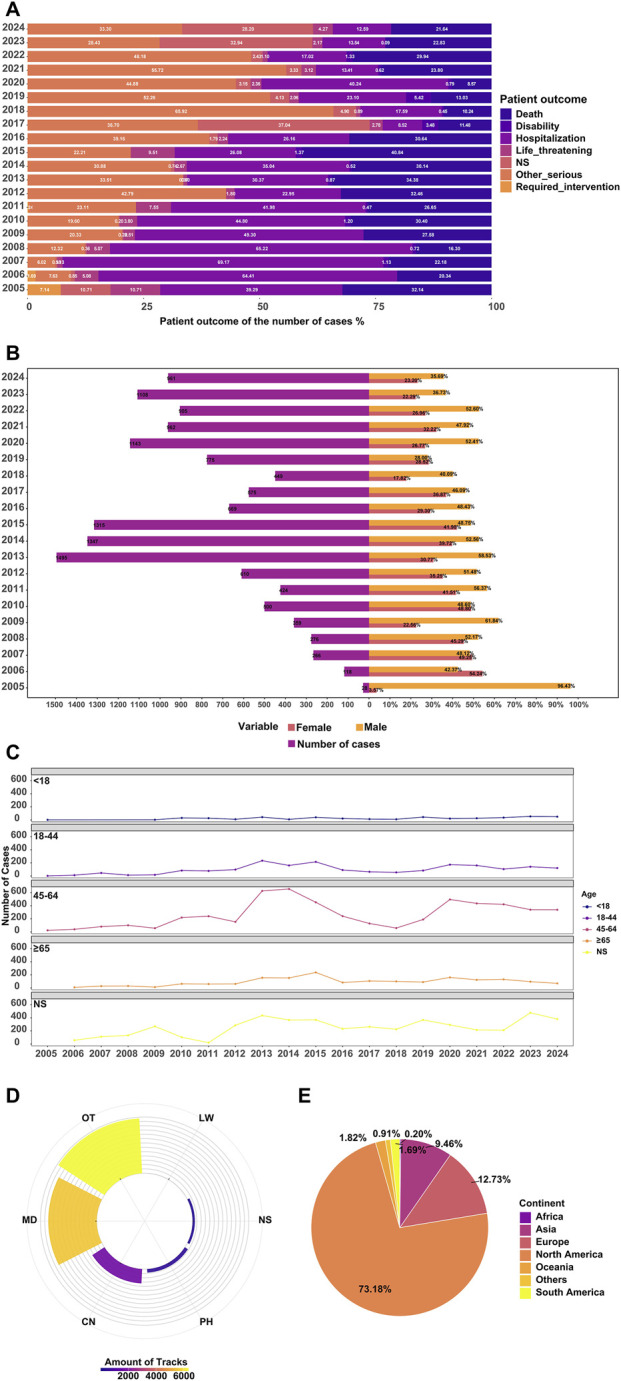
Demographic Characteristics of BEV ADE Reports. Note: **(A)** Annual distribution of adverse event outcomes; **(B)** Sex distribution by year (counts); **(C)** Age distribution by year (counts); **(D)** Reporter types (CN = consumer, LW = lawyer, SALES = salesperson, PH = pharmacist, MD = physician, RN = registered nurse, OT = other health professional, NS = not specified). Reporters were grouped into consumers (CN, LW, SALES) and healthcare professionals (RN, PH, MD, OT); NS denotes missing data; **(E)** Geographic distribution of reports by country/region.

Between the first quarter of 2004 and the fourth quarter of 2024, a total of 14,285 AE reports related to BEV in glioma patients were collected (Figures 3A–E). The temporal trend (Figure 3A), the annual number of bevacizumab-related reports increased steadily from 2005 to 2013, with an average yearly growth of 22.3%, broadly paralleling the period around FDA approval of bevacizumab for recurrent glioblastoma in 2009. In 2014, the number of reports declined by approximately 38.7%, temporally coinciding with the issuance of safety warnings, although the spontaneous nature of FAERS reporting precludes any inference of causality. Thereafter, from 2020 to 2024, reporting volumes remained largely stable (year-to-year fluctuations within ±5%), potentially reflecting the maturation of prescribing patterns and pharmacovigilance systems. The gender distribution (Figure 3B; Table 4) indicates that male patients accounted for 48.0% of the reports, exceeding females at 32.4%, while 19.6% were missing. Age distribution (Figure 3C; Table 4) shows the highest proportion in the 45–64 age group (37.1%), followed by 18–44 years (13.7%) and ≥65 years (12.4%), with the <18 group lowest at 3.1%; 33.7% of records lacked age information. In the geographic distribution (Figure 3D), reports from North America accounted for 73.2%, markedly exceeding those from Europe (12.7%), Asia (9.5%), South America (1.7%), Oceania (1.8%) and Africa (0.2%) (χ^2^ = 348.5, *p* < 0.001). This disparity likely reflects not only differences in regional pharmacovigilance systems (e.g., MedWatch in the United States and EudraVigilance in Europe), but also regulatory heterogeneity: bevacizumab was approved by the FDA in 2009 for recurrent glioblastoma, whereas the European Medicines Agency did not grant this indication, potentially contributing to higher use and reporting in North America. This disparity may reflect the mandatory MedWatch reporting system in the United States and the use of alternative systems such as EudraVigilance in Europe. RPSR (Figure 3E) were dominated by OT (44.4%) and MD (39.2%), followed by CN (11.7%) and PH (2.9%), with smaller contributions from nurses (RN) and 1.8% missing (NS). Clinical outcomes (Figure 3A; Table 4) were primarily other serious AEs (35.6%), HO (27.5%), and DE (25.4%), while LT (3.2%), DS (1.0%), and RI (0.0%) were infrequent; 7.2% were missing. The mean time to onset was 97.53 days (SD 180.15), indicating that while many events occurred early in treatment, delayed onset remained a notable risk.

**TABLE 4:** Clinical characteristics of bevacizumab reports in glioma patients in the FAERS database.

​	Level	Overall
n	​	14,285
Sex (%)	F	4,623 (32.4)
​	M	6,863 (48.0)
​	NS	2,799 (19.6)
Age (%)	<18	441 (3.1)
​	18–44	1953 (13.7)
​	45–64	5,306 (37.1)
​	≥65	1772 (12.4)
​	NS	4,813 (33.7)
Weight (%)	<80	3,782 (26.5)
​	≥80	3,320 (23.2)
​	NS	7,183 (50.3)
Year (%)	2005	28 (0.2)
​	2006	118 (0.8)
​	2007	266 (1.9)
​	2008	276 (1.9)
​	2009	359 (2.5)
​	2010	500 (3.5)
​	2011	424 (3.0)
​	2012	610 (4.3)
​	2013	1,495 (10.5)
​	2014	1,347 (9.4)
​	2015	1,315 (9.2)
​	2016	669 (4.7)
​	2017	575 (4.0)
​	2018	449 (3.1)
​	2019	775 (5.4)
​	2020	1,143 (8.0)
​	2021	962 (6.7)
​	2022	905 (6.3)
​	2023	1,108 (7.8)
​	2024	961 (6.7)
SOC (%)	BLOOD AND LYMPHATIC SYSTEM DISORDERS	622 (4.4)
​	CARDIAC DISORDERS	148 (1.0)
​	CONGENITAL, FAMILIAL AND GENETIC DISORDERS	3 (0.0)
​	EAR AND LABYRINTH DISORDERS	24 (0.2)
​	ENDOCRINE DISORDERS	21 (0.1)
​	EYE DISORDERS	172 (1.2)
​	GASTROINTESTINAL DISORDERS	1,180 (8.3)
​	GENERAL DISORDERS AND ADMINISTRATION SITE CONDITIONS	2,488 (17.4)
​	HEPATOBILIARY DISORDERS	124 (0.9)
​	IMMUNE SYSTEM DISORDERS	40 (0.3)
​	INFECTIONS AND INFESTATIONS	692 (4.8)
​	INJURY, POISONING AND PROCEDURAL COMPLICATIONS	1,048 (7.3)
​	INVESTIGATIONS	1,669 (11.7)
​	METABOLISM AND NUTRITION DISORDERS	339 (2.4)
​	MUSCULOSKELETAL AND CONNECTIVE TISSUE DISORDERS	499 (3.5)
​	NEOPLASMS BENIGN, MALIGNANT AND UNSPECIFIED (INCL CYSTS AND POLYPS)	760 (5.3)
​	NERVOUS SYSTEM DISORDERS	1934 (13.5)
​	NS	61 (0.4)
​	PREGNANCY, PUERPERIUM AND PERINATAL CONDITIONS	3 (0.0)
​	PRODUCT ISSUES	6 (0.0)
​	PSYCHIATRIC DISORDERS	417 (2.9)
​	RENAL AND URINARY DISORDERS	268 (1.9)
​	REPRODUCTIVE SYSTEM AND BREAST DISORDERS	8 (0.1)
​	RESPIRATORY, THORACIC AND MEDIASTINAL DISORDERS	648 (4.5)
​	SKIN AND SUBCUTANEOUS TISSUE DISORDERS	364 (2.5)
​	SOCIAL CIRCUMSTANCES	31 (0.2)
​	SURGICAL AND MEDICAL PROCEDURES	45 (0.3)
​	VASCULAR DISORDERS	671 (4.7)
Occupation of the reporter (%)	CN	1,666 (11.7)
​	LW	3 (0.0)
​	MD	5,596 (39.2)
​	NS	262 (1.8)
​	OT	6,346 (44.4)
​	PH	412 (2.9)
Continent of the reporter (%)	Africa	29 (0.2)
​	Asia	1,352 (9.5)
​	Europe	1818 (12.7)
​	North America	10,454 (73.2)
​	Oceania	260 (1.8)
​	Others	130 (0.9)
​	South America	242 (1.7)
Outcome (%)	DE	3,633 (25.4)
​	DS	143 (1.0)
​	HO	3,935 (27.5)
​	LT	453 (3.2)
​	NS	1,024 (7.2)
​	OT	5,092 (35.6)
​	RI	5 (0.0)
Time to onset (mean (SD))	​	97.53 (180.15)

Data source FAERS, database, 2004Q1–2024Q4.

### Time of AE occurrence

**FIGURE 4:**
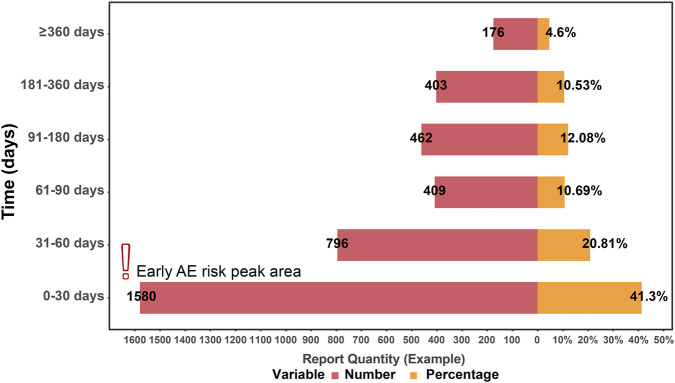
Time distribution of Bevacizumab-related adverse events (n = 3,826). Note: Only reports with available time-to-onset information were included (26.8% of all reports). Red bars indicate the number of reports (n), and orange bars indicate percentages (%). Time-to-onset was defined as the interval in days (d) between the event date and treatment start date. AE = adverse event. The “Early AE risk peak area” is a visual aid highlighting the early risk peak region and does not represent an additional data variable.

Among the 14,285 AE reports, only 3,826 (26.8%) documented the timing of AEs (Figure 4). Overall, the temporal distribution reveals a pronounced early risk peak: 41.3% (n = 1,580) of AEs occurred within 0–30 days post-medication, a significantly higher proportion compared to other time intervals (χ^2^ test, *p* < 0.001).

In terms of specific time intervals, the incidence from 31 to 60 days was 20.81% (n = 796), from 61 to 90 days was 10.69% (n = 409), from 91 to 180 days was 12.08% (n = 462), from 181 to 360 days was 10.53% (n = 403), while late-onset events occurring ≥360 days accounted for 4.6% (n = 176).

It is noteworthy that 73.2% of the reports lacked time information, primarily due to: (1) incomplete recording of event dates during emergency treatment; and (2) interrupted follow-up in patients with long-term therapy. This lack of data may lead to an underestimation or obscuration of long-term AE risks, particularly those occurring ≥360 days.

### AE reports and corresponding SOC signal composition ratios

**FIGURE 5:**
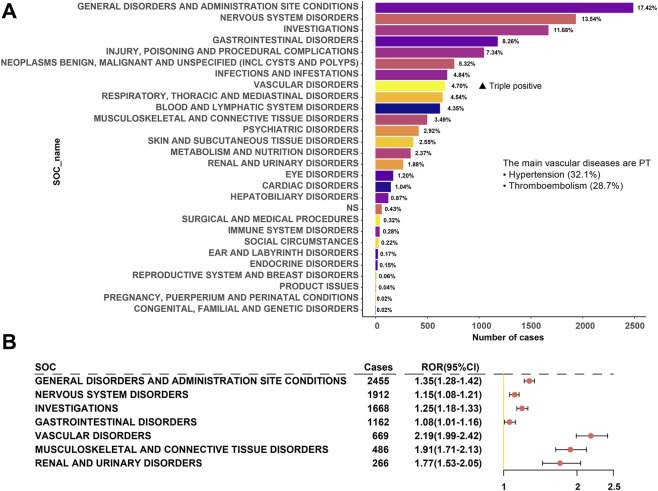
AE Events Related to BEV at the SOC Level in the FAERS Database. Note: **(A)** Distribution of case numbers across 27 SOC categories, sorted in descending order by case count; **(B)** Forest plot of positive ROR for the top seven SOCs. SOC = System Organ Class; AE = Adverse Event; ROR = Reporting Odds Ratio; PRR = Proportional Reporting Ratio; IC_025_ = Lower Bound of Bayesian CI. A positive signal is defined as ROR >1 with a 95% CI lower bound >1; consistent positivity across methods indicates that ROR, PRR, and IC_025_ all meet the criteria for a positive signal.

BEV-related AEs were associated with 27 SOCs (Figures 5A,B). The top seven SOCs by case count were as follows: general disorders and administration site conditions (n = 2,455, ROR = 1.35, 95% CI 1.28–1.42), nervous system disorders (n = 1912, ROR = 1.15, 95% CI 1.08–1.21), investigations (n = 1,668, ROR = 1.25, 95% CI 1.18–1.33), gastrointestinal disorders (n = 1,162, ROR = 1.08, 95% CI 1.01–1.16), vascular disorders (n = 669, ROR = 2.19, 95% CI 1.99–2.42), musculoskeletal and connective tissue disorders (n = 486, ROR = 1.91, 95% CI 1.71–2.13), and renal and urinary disorders (n = 266, ROR = 1.77, 95% CI 1.53–2.05).

In terms of signal strength (Figure 5B), vascular disorders emerged as the sole SOC category consistently positive across all three signal detection methods (ROR >1.99, PRR = 2.14, IC_025_ = 0.65), with significantly higher metrics than other systems (e.g., musculoskeletal and connective tissue disorders ROR = 1.91; renal and urinary disorders ROR = 1.77). Notably, although nervous system disorders were the second most reported, their signal strength was the lowest (ROR = 1.15), suggesting potential confounding factors related to primary diseases. Within the vascular disorders category, hypertension (32.1%), thromboembolism (28.7%), and hemorrhagic events (19.3%) were the predominant types.

### Signal characteristics and time distribution of BEV-Related AEs at the PT level in the FAERS database

**FIGURE 6:**
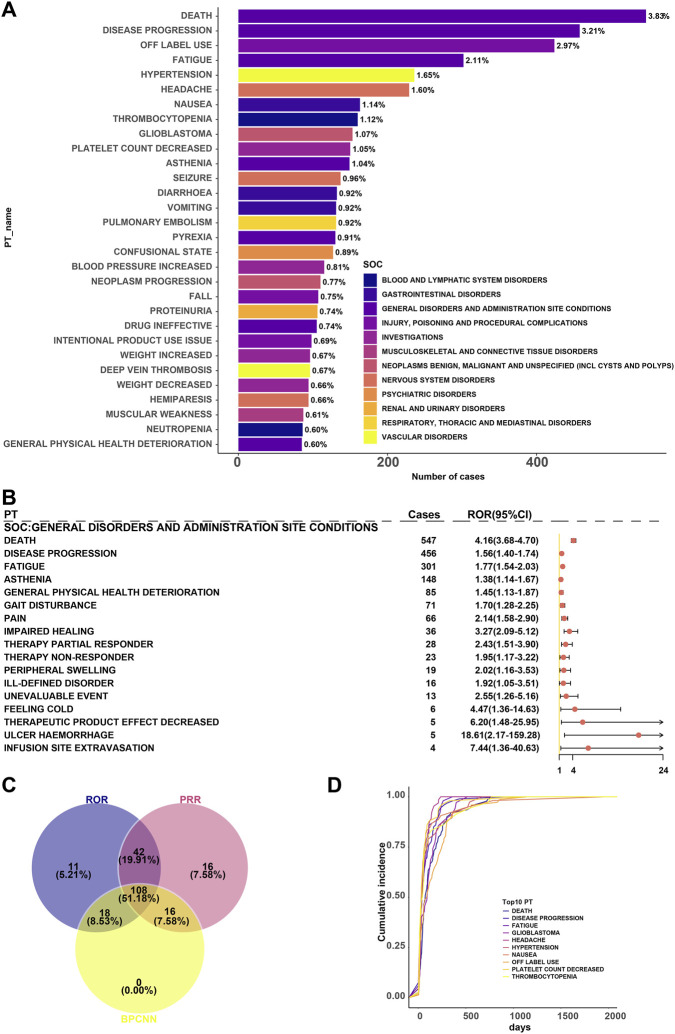
Signal Strength Analysis of BEV-Related AEs at the PT Level in the FAERS Database. Note: **(A)** Reporting frequency of the top 30 PTs and their associated SOC; **(B)** Forest plot of positive ROR for selected high-frequency PTs; **(C)** Venn diagram showing the number of PTs significantly positive across three methods (core risk area); **(D)** Cumulative incidence trends of the top 10 most frequently reported PTs in the FAERS database, with the x-axis representing report years and the y-axis representing cumulative incidence (%). Data source: FAERS database (2004Q1-2024Q4).

In the FAERS database, we conducted a disproportionality analysis of AEs related to BEV in glioma patients at the PT level. Signal strength was assessed using three methods: ROR, PRR, and BCPNN. We identified 179 PTs as positive in the ROR analysis, 182 PTs in the PRR analysis, and 142 PTs in the BCPNN analysis. Among these, 108 PTs were significantly positive across all three methods, which can be considered core risk signals (Figures 6A,C).

Among the 30 most frequently reported PTs (Table 5), many represent known or reasonably explained AEs associated with BEV. For example, hypertension (n = 234, ROR = 5.04 [4.14–6.14], PRR = 4.97 [319.87], IC_025_ = 1.43 [1.20]), increased blood pressure (n = 115, ROR = 5.38 [4.04–7.17], PRR = 5.35 [167.24], IC_025_ = 1.48 [1.14]), and proteinuria (n = 106, ROR = 11.68 [7.93–17.19], PRR = 11.60 [249.81], IC_025_ = 1.84 [1.47]) all exhibited high signal strength, indicating a strong association with BEV’s mechanism of inhibiting angiogenesis and damaging the glomerular filtration barrier.

**TABLE 5:** Top 30 preferred terms (PTs) associated with bevacizumab ranked by reporting frequency.

SOC	PT	Number of reports(a)	b	c	d	ROR	ROR_L	ROR_U	ROR (95%CI)	PRR	Chi_Square	PRR (Chi_Square)	IC	IC025	IC(IC025)
GENERAL DISORDERS AND ADMINISTRATION SITE CONDITIONS	DEATH	547	13,548	504	51,929	4.16	3.68	4.7	4.16 (3.68–4.70)	4.04	609.01	4.04 (609.01)	1.3	1.14	1.30 (1.14)
GENERAL DISORDERS AND ADMINISTRATION SITE CONDITIONS	DISEASE PROGRESSION	456	13,639	1,101	51,332	1.56	1.4	1.74	1.56 (1.40–1.74)	1.54	62.65	1.54 (62.65)	0.47	0.31	0.47 (0.31)
INJURY, POISONING AND PROCEDURAL COMPLICATIONS	OFF LABEL USE	422	13,673	936	51,497	1.7	1.51	1.91	1.70 (1.51–1.91)	1.68	81.18	1.68 (81.18)	0.55	0.39	0.55 (0.39)
GENERAL DISORDERS AND ADMINISTRATION SITE CONDITIONS	FATIGUE	301	13,794	640	51,793	1.77	1.54	2.03	1.77 (1.54–2.03)	1.75	66.68	1.75 (66.68)	0.59	0.4	0.59 (0.40)
VASCULAR DISORDERS	HYPERTENSION	234	13,861	175	52,258	5.04	4.14	6.14	5.04 (4.14–6.14)	4.97	319.87	4.97 (319.87)	1.43	1.2	1.43 (1.20)
NERVOUS SYSTEM DISORDERS	HEADACHE	216	13,879	513	51,920	1.58	1.34	1.85	1.58 (1.34–1.85)	1.57	31.47	1.57 (31.47)	0.48	0.26	0.48 (0.26)
GASTROINTESTINAL DISORDERS	NAUSEA	161	13,934	638	51,795	0.94	0.79	1.12	0.94 (0.79–1.12)	0.94	0.52	0.94 (0.52)	−0.07	−0.32	−0.07 (-0.32)
BLOOD AND LYMPHATIC SYSTEM DISORDERS	THROMBOCYTOPENIA	160	13,935	1,015	51,418	0.58	0.49	0.69	0.58 (0.49–0.69)	0.59	41.04	0.59 (41.04)	−0.64	−0.88	−0.64 (-0.88)
NEOPLASMS BENIGN, MALIGNANT AND UNSPECIFIED (INCL CYSTS AND POLYPS)	GLIOBLASTOMA	153	13,942	312	52,121	1.83	1.51	2.23	1.83 (1.51–2.23)	1.82	38.5	1.82 (38.50)	0.64	0.37	0.64 (0.37)
INVESTIGATIONS	PLATELET COUNT DECREASED	150	13,945	393	52,040	1.42	1.18	1.72	1.42 (1.18–1.72)	1.42	13.59	1.42 (13.59)	0.38	0.12	0.38 (0.12)
GENERAL DISORDERS AND ADMINISTRATION SITE CONDITIONS	ASTHENIA	148	13,947	400	52,033	1.38	1.14	1.67	1.38 (1.14–1.67)	1.38	11.21	1.38 (11.21)	0.35	0.08	0.35 (0.08)
NERVOUS SYSTEM DISORDERS	SEIZURE	137	13,958	518	51,915	0.98	0.81	1.19	0.98 (0.81–1.19)	0.98	0.03	0.98 (0.03)	−0.02	−0.29	−0.02 (-0.29)
RESPIRATORY, THORACIC AND MEDIASTINAL DISORDERS	PULMONARY EMBOLISM	130	13,965	449	51,984	1.08	0.89	1.31	1.08 (0.89–1.31)	1.08	0.56	1.08 (0.56)	0.08	−0.2	0.08 (-0.20)
GASTROINTESTINAL DISORDERS	DIARRHOEA	129	13,966	434	51,999	1.11	0.91	1.35	1.11 (0.91–1.35)	1.11	1.01	1.11 (1.01)	0.11	−0.17	0.11 (-0.17)
PSYCHIATRIC DISORDERS	CONFUSIONAL STATE	127	13,968	341	52,092	1.39	1.13	1.7	1.39 (1.13–1.70)	1.39	9.99	1.39 (9.99)	0.36	0.07	0.36 (0.07)
GASTROINTESTINAL DISORDERS	VOMITING	119	13,976	603	51,830	0.73	0.6	0.89	0.73 (0.60–0.89)	0.73	9.68	0.73 (9.68)	−0.36	−0.65	−0.36 (-0.65)
GENERAL DISORDERS AND ADMINISTRATION SITE CONDITIONS	PYREXIA	118	13,977	678	51,755	0.64	0.53	0.78	0.64 (0.53–0.78)	0.65	19.53	0.65 (19.53)	−0.52	−0.8	−0.52 (-0.80)
INVESTIGATIONS	BLOOD PRESSURE INCREASED	115	13,980	80	52,353	5.38	4.04	7.17	5.38 (4.04–7.17)	5.35	167.24	5.35 (167.24)	1.48	1.14	1.48 (1.14)
NEOPLASMS BENIGN, MALIGNANT AND UNSPECIFIED (INCL CYSTS AND POLYPS)	NEOPLASM PROGRESSION	110	13,985	377	52,056	1.09	0.88	1.34	1.09 (0.88–1.34)	1.09	0.58	1.09 (0.58)	0.09	−0.21	0.09 (-0.21)
INJURY, POISONING AND PROCEDURAL COMPLICATIONS	FALL	107	13,988	303	52,130	1.32	1.05	1.64	1.32 (1.05–1.64)	1.31	5.96	1.31 (5.96)	0.3	−0.01	0.30 (-0.01)
RENAL AND URINARY DISORDERS	PROTEINURIA	106	13,989	34	52,399	11.68	7.93	17.19	11.68 (7.93–17.19)	11.6	249.81	11.60 (249.81)	1.84	1.47	1.84 (1.47)
GENERAL DISORDERS AND ADMINISTRATION SITE CONDITIONS	DRUG INEFFECTIVE	105	13,990	622	51,811	0.63	0.51	0.77	0.63 (0.51–0.77)	0.63	20.02	0.63 (20.02)	−0.55	−0.85	−0.55 (-0.85)
INJURY, POISONING AND PROCEDURAL COMPLICATIONS	INTENTIONAL PRODUCT USE ISSUE	98	13,997	155	52,278	2.36	1.83	3.04	2.36 (1.83–3.04)	2.35	46.84	2.35 (46.84)	0.87	0.53	0.87 (0.53)
INVESTIGATIONS	WEIGHT INCREASED	96	13,999	127	52,306	2.82	2.17	3.68	2.82 (2.17–3.68)	2.81	64.05	2.81 (64.05)	1.02	0.67	1.02 (0.67)
VASCULAR DISORDERS	DEEP VEIN THROMBOSIS	96	13,999	284	52,149	1.26	1	1.59	1.26 (1.00–1.59)	1.26	3.8	1.26 (3.80)	0.25	−0.07	0.25 (-0.07)
INVESTIGATIONS	WEIGHT DECREASED	94	14,001	187	52,246	1.88	1.46	2.4	1.88 (1.46–2.40)	1.87	25.42	1.87 (25.42)	0.66	0.32	0.66 (0.32)
NERVOUS SYSTEM DISORDERS	HEMIPARESIS	92	14,003	274	52,159	1.25	0.99	1.59	1.25 (0.99–1.59)	1.25	3.44	1.25 (3.44)	0.25	−0.09	0.25 (-0.09)
MUSCULOSKELETAL AND CONNECTIVE TISSUE DISORDERS	MUSCULAR WEAKNESS	87	14,008	189	52,244	1.72	1.33	2.21	1.72 (1.33–2.21)	1.71	17.73	1.71 (17.73)	0.57	0.22	0.57 (0.22)
BLOOD AND LYMPHATIC SYSTEM DISORDERS	NEUTROPENIA	86	14,009	563	51,870	0.57	0.45	0.71	0.57 (0.45–0.71)	0.57	24.72	0.57 (24.72)	−0.68	−1.01	−0.68 (-1.01)
GENERAL DISORDERS AND ADMINISTRATION SITE CONDITIONS	GENERAL PHYSICAL HEALTH DETERIORATION	85	14,010	218	52,215	1.45	1.13	1.87	1.45 (1.13–1.87)	1.45	8.59	1.45 (8.59)	0.4	0.05	0.40 (0.05)

Data source FAERS, database, 2004Q1–2024Q4.

Additionally, some events, though not prominently highlighted in prescribing information, exhibited notable reporting frequency and signal strength, such as product use issue (n = 98, ROR = 2.36 [1.83–3.04], PRR = 2.35 [46.84], IC_025_ = 0.87 [0.53]) and weight increased (n = 96, ROR = 2.82 [2.17–3.68], PRR = 2.81 [64.05], IC_025_ = 1.02 [0.67]).

DE (n = 547, ROR = 4.16 [3.68–4.70], PRR = 4.04 [609.01], IC_025_ = 1.30 [1.14]) was the most frequently reported PT. After excluding cases associated with disease progression, the signal strength decreased by approximately 37%, suggesting a partial association with the natural history of the disease (Figure 6B). To further illustrate the occurrence patterns of these high-frequency AEs, we plotted the cumulative incidence changes of the top 10 most frequently reported PTs during the study period, indicating that most AEs reached high proportions early in the treatment and then stabilized (Figure 6D).

### Impact of demographic characteristics and reporter variability

To investigate the impact of demographic characteristics and reporter type on signals related to BEV AEs, we conducted a disproportionality analysis based on the FAERS database, stratified by gender, age, and reporter type. A Cochran-Mantel-Haenszel interaction test (*p*-interaction <0.05) was employed to assess heterogeneity. Within each subgroup, only PTs meeting the criteria for a positive ROR (lower limit of 95% CI > 1) were retained. Consistency was evaluated by integrating results from PRR and BCPNN, and signals positive across all three methods were defined as core risk signals.

**FIGURE 7:**
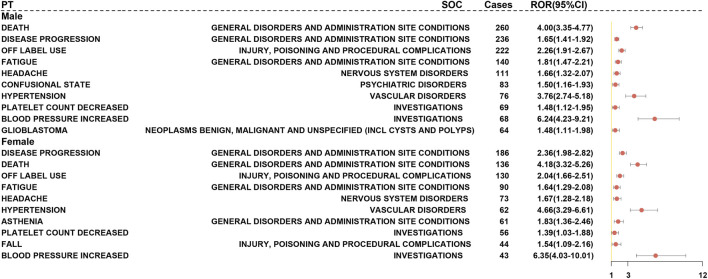
Forest Plot of BEV-Related AE Positive Signals Stratified by Gender (FAERS Database). Note: Stratified disproportionality analysis based on the Cochran–Mantel–Haenszel test (heterogeneity defined as p-interaction <0.05), displaying prespecified mechanism-related core PTs (hypertension, proteinuria, bleeding, thrombosis) and other significant signals. Only ROR estimates that remained significant after Benjamini–Hochberg correction (q < 0.05) are shown; dots indicate ROR values and horizontal bars represent 95% confidence intervals. PTs with labels denote concordant positive signals across all three methods (ROR, PRR and BCPNN).

Core risk signals detected in both male and female patients included DE, off-label use, hypertension, increased blood pressure, weight loss, and weight gain (Figure 7). Notably, hypertension (male: ROR = 3.14 [2.76–3.56]; female: ROR = 3.68 [3.22–4.20]) and proteinuria (male: ROR = 10.54 [7.89–14.07]; female: ROR = 12.13 [8.92–16.48]) were significantly positive across all three methods, suggesting a strong association with BEV’s angiogenesis inhibition mechanism. Male-specific positive signals included bleeding (ROR = 3.20 [2.10–4.80]), increased diastolic pressure (ROR = 2.80 [1.90–4.10]), dizziness, depression, arthralgia, and attention disorder. Female-specific signals encompassed disease progression, gait disturbance, cerebral hemorrhage (ROR = 4.10 [2.90–5.80]), cognitive impairment (ROR = 3.60 [2.60–5.00]), increased heart rate, and unspecified diseases. Notably, disproportionality signals for overall bleeding were stronger in males, whereas females exhibited stronger signals for severe bleeding phenotypes, particularly intracranial hemorrhage, a pattern that may reflect differences in event grading, sample size and sex-related biology. Among pharmacologically mediated adverse events, signals for hypertension, proteinuria, bleeding and thromboembolic events were the most prominent. By contrast, terms such as “death”, “disease progression”, “off-label use” and “product use issue” were treated as use- or outcome-related events and reported separately for descriptive context.

**FIGURE 8:**
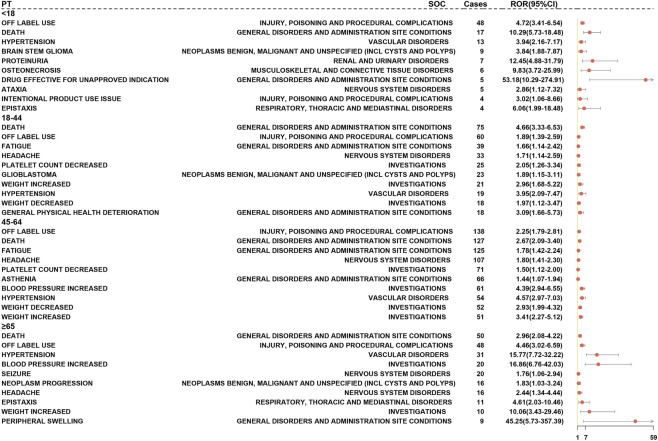
Forest Plot of BEV-Related AE Positive Signals Stratified by Age (FAERS Database). Note: The analytical method is consistent with that in Figure 7; Core PTs are positive across all age groups, while specific events appear exclusively in their respective age groups. Analyses applied a consistent statistical strategy, using the Cochran-Mantel-Haenszel test to assess heterogeneity and the Benjamini–Hochberg procedure to control for multiplicity.

Core risk signals identified across all four age groups prominently included proteinuria and hypertension, both exhibiting high signal strength (proteinuria ROR range: 9.85–12.67; hypertension ROR range: 2.94–4.12). The 45–64 age group was characterized by a significant number of high-risk events, including thrombosis (ROR = 5.10 [3.20–8.20]) and muscle cramps (ROR = 3.70 [2.40–5.80]). In patients aged 65 and above, there was a marked increase in vascular toxicity, with a notably higher reporting rate of hypertension (*p* < 0.01) (Figure 8). These findings underscore the necessity of monitoring and preventing vascular-related REAC in middle-aged and elderly patients undergoing BEV treatment.

**FIGURE 9:**
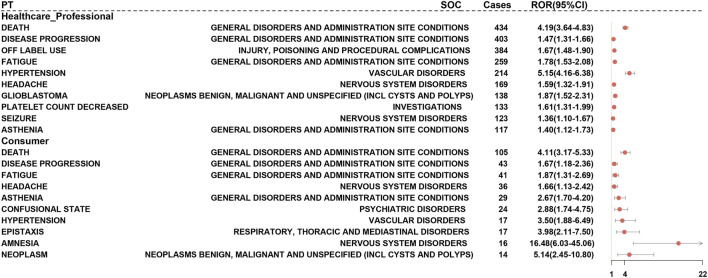
Forest Plot of BEV-Related AE Signals Stratified by Reporter Category (FAERS Database). Note: The analysis method is consistent with Figure 7; unique signals for subgroups of medical professionals and CN-related reporters are specifically highlighted, with core PT representing events that are positive in all three methods. Analyses applied a consistent statistical strategy, using the Cochran-Mantel-Haenszel test to assess heterogeneity and the Benjamini–Hochberg procedure to control for multiplicity”.

In the subgroups of healthcare professionals and CN-related reporters, core risk signals included DE, headache, fatigue, increased blood pressure, and proteinuria (Figure 9). Signals specific to healthcare professionals were concentrated on hematological and tumor-related events, such as lymphocyte reduction (ROR = 5.14 [2.50–10.80]) and malignant glioma (ROR = 3.87 [1.90–7.90]), indicating their proficiency in identifying rare yet severe AEs. In contrast, the CN-related reporters subgroup reported relatively higher instances of nervous system events, such as amnesia and aphasia, though the wide CIs reflect the instability of results from smaller samples.

In summary, subgroup analysis reveals differences in the distribution of BEV-related AEs across gender, age, and reporter categories. Certain core PTs consistently exhibited strong signals across multiple analytical methods, indicating their importance as monitoring priorities.

## Discussion

Using FAERS data from 2004Q1 to 2024Q4, we systematically characterized 14,285 adverse event reports for bevacizumab in patients with glioma. Bevacizumab-related events spanned 27 system organ classes, with particularly prominent signals for vascular disorders, hypertension, proteinuria and thromboembolic events, while terms such as “death”, “disease progression”, “off-label use” and “product use issue” were treated as use- or outcome-related and reported descriptively rather than as primary pharmacologic signals. A substantial proportion of adverse events clustered within the first month of exposure, whereas late-onset events, including tumor progression, also contributed meaningfully, underscoring the need for both early vigilance and longer-term follow-up, although time-to-onset analyses were constrained by missing onset dates in most reports. Subgroup analyses suggested higher overall bleeding and thrombotic signals in males and a greater burden of severe bleeding phenotypes such as intracranial hemorrhage in females, alongside a peak adverse event burden in patients aged 45–64 years, consistent with known pharmacology and prior observations in anti-angiogenic therapy.

North America contributed the majority of reports, which likely reflects not only differences in pharmacovigilance infrastructure but also regulatory divergence, given that bevacizumab has been approved for recurrent glioblastoma by the FDA since 2009, whereas the EMA has not granted this indication. Although FAERS does not permit causal inference or precise estimation of absolute risks, it provides a useful lens on real-world safety signals and utilization patterns. Compared with previous analyses based on VigiBase, EudraVigilance or single clinical cohorts (Smolenschi et al., 2023; Oikawa et al., 2022), the present study covers a longer time span, a larger sample and integrates multiple signal-detection methods with stratified analyses, thereby extending pharmacovigilance evidence on bevacizumab. These findings should be interpreted as hypothesis-generating rather than causal, particularly in the context of frequent combination therapy (e.g., temozolomide, corticosteroids) and disease-related vascular and renal complications, which may confound the observed signals and warrant confirmation in prospective studies.

In comparison with previous studies, the findings of this study align closely with the results of several RCTs. For instance, both the AVAglio and RTOG0825 trials reported higher incidences of hypertension, thrombosis, and proteinuria associated with BEV treatment (Wang et al., 2024; Funakoshi et al., 2020), which are corroborated by the strong signals for these core AEs in our study. Additionally, we observed that the relative burden of vascular-related events in real-world settings may exceed that reported in RCTs, highlighting the significant impact of comorbidity complexity and differences in clinical practice environments on risk assessment. Furthermore, our study identified signals such as cognitive impairment and weight gain, which are rarely mentioned in clinical trials, indicating that real-world data can Supplementary Material on infrequent or atypical AEs. Endothelial dysfunction and glomerular injury induced by VEGF inhibition may explain the risks of hypertension and proteinuria. The coexistence of thrombosis and hemorrhage could be related to a coagulation-fibrinolysis imbalance and tumor-associated hypercoagulable states (Chen et al., 2025; Robinson et al., 2023).

To validate the robustness of the safety signals identified from the FAERS data in this study, we compared our findings with multiple recent pharmacovigilance analyses of bevacizumab conducted using EudraVigilance and VigiBase over the past 5 years (Table 6). Most of these studies employed disproportionality analyses and consistently highlighted hypertension, proteinuria, and thromboembolic/embolic events as predominant high-signal adverse reactions. For instance, Vonica et al. reported that serious adverse reactions accounted for as much as 93.74% of cases in EudraVigilance, with a proteinuria signal of comparable strength to that observed in our study (Vonica et al., 2025). Similarly, Ishizawa et al., using VigiBase, identified hypertension (ROR = 5.11) and proteinuria (ROR = 9.87) as the principal risk signals (Niimura et al., 2023). Wang et al., analyzing OpenVigil-FAERS, also confirmed the significance of multiple hemorrhagic, thrombotic, and perforation-related signals (Wang et al., 2024). In addition, Tang et al. focused on bevacizumab-associated bleeding events, identifying 37 statistically significant high-signal adverse reactions in FAERS, underscoring their severity and lethality (Tang et al., 2023). Hoeger et al., integrating VigiBase with TriNetX cohorts, confirmed the cardiotoxicity risk of bevacizumab combined with PLD, particularly regarding hypertension and pericardial disease (Hoeger et al., 2025). Likewise, Hu et al. demonstrated that pulmonary hemorrhage and hemoptysis signals were stronger, and mortality higher, in patients receiving bevacizumab-based chemotherapy regimens (Hu et al., 2024b). Collectively, these findings closely align with our results, providing external corroboration of the reliability and reproducibility of the safety signals identified in this study.

**TABLE 6:** Comparative analysis of bevacizumab-related adverse events in different databases.

Author (Year)	Database source	Study period	Total AEs	Proportion of serious ADRs	Main high-signal adverse reactions*	Remarks
FAERS (this study)	US FAERS	2004Q1–2024Q4	14,285	68.40%	Hypertension (ROR = 5.04, 95%CI 4.80–5.28); Proteinuria (ROR = 11.68, 95%CI 10.92–12.49); Vascular events (ROR = 2.19, 95%CI 1.99–2.42); Pulmonary embolism (ROR = 3.87, 95%CI 3.52–4.25)	Subgroup analysis revealed significant differences by sex and age
Vonica et al. (2025)	EudraVigilance	2005–2023	8,412	93.74%	Hypertension (ROR = 4.92); Thrombosis/embolism (ROR = 3.65); Proteinuria (ROR = 10.24)	Quantitative description + disproportionality analysis
Niimura et al., (2023)	VigiBase	2009–2021	12,307	72.10%	Hypertension (ROR = 5.11); Hemorrhagic events (ROR = 2.98); Proteinuria (ROR = 9.87); Gastrointestinal perforation (ROR = 4.26)	Comparative analysis across multiple drugs
Wang et al. (2024)	US FAERS	2017–2022	21,161	Not explicitly stated	Hypertension (PRR = 6.51); Proteinuria (PRR = 37.16); Hemorrhage (PRR = 2.66); Arterial thromboembolism (PRR = 6.75); Gastrointestinal perforation (PRR = 90.46); Nasal septum perforation (PRR = 47.50); Necrotizing fasciitis (PRR = 20.26); Hypertensive encephalopathy (PRR = 18.29)	Signal detection based on OpenVigil-FDA; combined analysis of FAERS and 8 RCTs; highlighted discrepancy between real-world signals and drug label warnings
Tang et al. (2023)	FAERS	2014Q1–2022Q3	96,477 drug-event pairs (2,579 hemorrhagic signals)	Not specified (hemorrhagic signals emphasized)	CNS hemorrhage, gastrointestinal bleeding, pulmonary hemorrhage, retinal hemorrhage, haematuria, epistaxis, tumor bleeding, vaginal hemorrhage	Pharmacovigilance study using PRR methodology; 37 hemorrhagic adverse events identified with statistically significant disproportionality; strong emphasis on severe/fatal outcomes
Hoeger et al. (2025)	VigiBase + TriNetX	Up to Q3 2022	VigiBase: 730 AEs; TriNetX: 2,388 matched patients	Not applicable (cohort and disproportionality analysis)	Hypertension (ROR = 6.05); Pericardial disorders (ROR = 3.67); Heart failure (ROR = 1.75)	Propensity score-matched cohort study supported by signal detection via VigiBase; focus on cardiotoxicity associated with PLD plus bevacizumab combination therapy
Hu et al. (2024a)	FAERS	2004Q1–2023Q1	55,184 total reports (497 for pulmonary hemorrhage/hemoptysis)	Fatality rate of 45.5% among pulmonary hemorrhage cases	Pulmonary hemorrhage (ROR = 4.19); Hemoptysis (ROR = 5.35 for BV + chemotherapy regimen)	Retrospective disproportionality study comparing monotherapy and combination regimens; BV + chemotherapy associated with stronger signal intensity and higher fatality rate

During the analysis, this study also identified some unexpected findings. For instance, certain AEs such as cognitive impairment and weight gain, although not highlighted as primary warning events in the drug’s labeling, exhibited notable signal strength in the FAERS data. Cognitive impairment may be associated with the role of VEGF in regulating neuronal function, as experimental studies have suggested that VEGF plays a role in synaptic plasticity and cognitive regulation (Wang et al., 2021; Maurer et al., 2020). Consequently, VEGF inhibition could potentially lead to neurological effects. The signal for weight gain might be linked to fluid retention or metabolic disturbances following BEV use, which has not been extensively reported in previous literature (Patil et al., 2021). The identification of these atypical AEs in this study suggests that the effects of BEV may extend beyond traditional angiogenesis inhibition. Compared to reports from some pharmacovigilance studies, this research particularly emphasizes the significance of these signals in the glioma population, warranting further investigation and validation in future studies.

From a clinical perspective, these findings have direct implications for practice. The prominent vascular and renal toxicities associated with bevacizumab in glioma underscore the need for vigilant monitoring of blood pressure, renal function and thrombotic risk during treatment. Males appear to be at higher risk of bleeding and thromboembolic events, warranting closer surveillance, although the role of prophylactic anticoagulation requires confirmation in prospective studies. Females more frequently reporting cognitive impairment suggests that longitudinal assessment of neurological function should be integrated into follow-up. Patients aged 45–64 years carry the greatest adverse event burden and may benefit from more intensive, individualized monitoring. In addition, the clustering of events early after treatment initiation supports designating the first month of therapy as a critical window for enhanced safety surveillance. Collectively, these observations inform risk stratification and signal validation, and provide a basis for future work on individualized safety management in bevacizumab-treated glioma.

Despite the broad coverage and large sample size of FAERS, this study has several limitations. First, FAERS is a spontaneous reporting system and is inherently subject to underreporting, overreporting and selection bias. Time-to-onset analyses relied on a non-random subset of reports with complete onset information; cases with more complete follow-up or more acute events are more likely to be reported, which may lead to overestimation of early events (0–30 days) and underestimation of late-onset risk. Second, reports frequently lack key clinical details, including exact dose, treatment duration, concomitant therapies and past medical history, and information on co-medications is often incomplete. As a result, we were unable to disentangle adverse events attributable to chemotherapy, corticosteroids or disease progression itself, which limits causal interpretation and confounder control. Third, disproportionality analyses capture statistical associations rather than causal effects, and the present findings should therefore be regarded as risk signals rather than definitive evidence of harm. Finally, subgroup analyses highlighting sex- and age-related differences are constrained by missing data and potential reporting biases and should be interpreted with caution.

Future work should address questions that could not be fully resolved in this analysis. First, unexpected signals such as cognitive impairment and weight gain require confirmation of causality and elucidation of underlying mechanisms through prospective clinical studies and experimental research. Second, multi-database and multicenter pharmacovigilance analyses integrating FAERS with other systems (e.g., VigiBase, EudraVigilance) are needed to enhance the robustness and generalizability of safety signals. Third, linkage with electronic health records and other real-world clinical datasets would provide richer information on treatment trajectories, co-medications and longitudinal outcomes, enabling more rigorous adjustment for confounding. Fourth, mechanistic studies should further explore the long-term impact of VEGF inhibition on neural function and metabolism to explain emerging safety signals. Finally, future research should focus on developing risk prediction models and individualized dosing and monitoring strategies to better balance efficacy and safety of bevacizumab in patients with glioma.

## Conclusion

**FIGURE 10:**
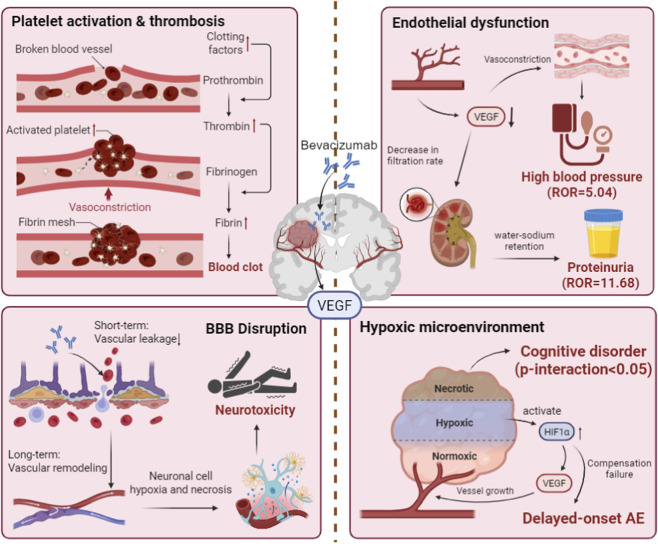
Mechanistic pathways of BEV-associated AE in glioma.

This study used the FAERS database to systematically characterize adverse events following bevacizumab use in patients with glioma, delineating its real-world safety profile. Bevacizumab-related events were predominantly clustered in vascular, renal/urinary and metabolism-related disorders, with clear heterogeneity across sex and age strata. In particular, strong signals were observed for vascular disorders, hypertension, proteinuria and thromboembolic events, underscoring the need for careful monitoring of these risks in clinical practice. The identification of specific high-risk subgroups, such as males aged 45–64 years, provides a basis for risk stratification and supports future precision medicine approaches. These findings highlight the importance of intensified pharmacovigilance in vulnerable populations (e.g., middle-aged and older males) and offer a signal-generating framework for subsequent studies on individualized risk mitigation, while optimal management strategies will require confirmation in prospective clinical research (Figure 10).

## Data Availability

The original contributions presented in the study are included in the article/Supplementary Material, further inquiries can be directed to the corresponding author.

## Glossary

AE: Adverse Event
BCPNN: Bayesian Confidence Propagation Neural Network
BEV: Bevacizumab
CA: Congenital Anomaly
CI: Confidence Interval
CN: Consumer
DE: Death
DEMO: Demographics and Administrative Information
DRUG: Drug Information
DS: Disability
FAERS: Food and Drug Administration Adverse Event Reporting System
FDA: Food and Drug Administration
GBM: Glioblastoma
HLGT: High-Level Group Terms
HLT: High-Level Terms
HO: Hospitalization
IC: Information Component
**IC**_**025**_: Information Component 025
INDI: Indications Information
IQR: Interquartile Range
LLT: Low-Level Terms
LT: Life-Threatening Events
LW: Lawyer
MD: Physician
NS: Not Specified
OS: Overall Survival
OT: Other Health Professional
OUTC: Patient Outcomes
PFS: Progression-Free Survival
PH: Pharmacist
PRR: Proportional Reporting Ratio
PS: Primary Suspect
PT: Preferred Terms
RCT: Randomized Controlled Trial
REAC: Adverse Reactions
RI: Required Intervention
RN: Registered Nurse
ROR: Reporting Odds Ratio
RPSR: Report Sources
SALES: Salesperson
SOCs: System Organ Classes
THER: Therapy Dates and Timing
VEGF: Vascular Endothelial Growth Factor
