# Supplementary material for: Real-world adverse event profile and signal characteristics of bevacizumab in glioma: a FAERS-based disproportionality analysis
Source: Front Pharmacol. 2026 Jan 13;16:1705451. doi: 10.3389/fphar.2025.1705451 (PMC12835385; doi:10.3389/fphar.2025.1705451)
Supplement: Supplementary file 1 [file Table1.docx]

**Table S1. MedDRA Preferred Terms (PTs) Corresponding to Glioma Diagnoses.**

| **No.** | **MedDRA Preferred Term (PT)** | **MedDRA Code** | **Classification (WHO CNS Classification)** |
| --- | --- | --- | --- |
| 1 | Ganglioglioma | 10017194 | Glioneuronal mixed tumour |
| 2 | Astrocytoma | 10003282 | Astrocytic tumour |
| 3 | Glioma | 10018392 | Glioma |
| 4 | Oligodendroglioma | 10030055 | Oligodendroglial tumour |
| 5 | Ependymoma | 10014527 | Ependymal tumour |
| 6 | Glioneuronal tumour | 10018396 | Glioneuronal mixed tumour |
| 7 | Angiocentric glioma | 10061131 | Angiocentric glioma |
| 8 | Astroblastoma | 10003284 | Astroblastoma |
| 9 | Malignant glioma | 10027859 | Malignant glioma |
| 10 | Glioblastoma multiforme | 10018395 | Glioblastoma multiforme |
| 11 | Glioblastoma | 10018393 | Glioblastoma |
| 12 | Anaplastic astrocytoma | 10002979 | Anaplastic astrocytoma |
| 13 | Oligoastrocytoma | 10030054 | Mixed oligoastrocytic tumour |
| 14 | Brain stem glioma | 10006442 | Brain stem glioma |
| 15 | Astrocytoma malignant | 10003283 | Malignant astrocytoma |
| 16 | Ependymoma malignant | 10014528 | Malignant ependymoma |
| 17 | Malignant oligodendroglioma | 10027860 | Malignant oligodendroglioma |
| 18 | Anaplastic oligodendroglioma | 10002980 | Anaplastic oligodendroglioma |
| 19 | Gliomatosis cerebri | 10018397 | Gliomatosis cerebri |
| 20 | Metastatic glioma | 10046013 | Metastatic glioma |
| 21 | Gliosarcoma | 10018398 | Gliosarcoma |
| 22 | Mixed oligo-astrocytoma | 10030056 | Mixed oligoastrocytic tumour |
| 23 | Mixed astrocytoma-ependymoma | 10061132 | Mixed astrocytic–ependymal tumour |
| 24 | Anaplastic ependymoma | 10002978 | Anaplastic ependymoma |
| 25 | Astrocytoma, low grade | 10003285 | Low-grade astrocytoma |
| 26 | Oligodendroglioma benign | 10030057 | Benign oligodendroglioma |
| 27 | Ependymoma benign | 10014529 | Benign ependymoma |
| 28 | Anaplastic ganglioglioma | 10002981 | Anaplastic ganglioglioma |
